# Endoscopic Interlaminar Decompression for T1-T2 Thoracic Disc Herniation: A Case Report

**DOI:** 10.7759/cureus.109875

**Published:** 2026-05-29

**Authors:** Abdulhakem Alshehri, Rakan M Khawaji, Turki B Alotaibi, Hussam Jabri, Emad Aldeen Mohamed

**Affiliations:** 1 Spine Surgery, King Fahad Military Medical Complex, Dhahran, SAU; 2 Orthopedic Surgery, Ministry of Health, Riyadh, SAU; 3 Spine Surgery, Prince Sultan Military Medical City, Riyadh, SAU

**Keywords:** disc prolapse, spinal disc herniation, thoracic disc, thoracic disc herniation, thoracic spine surgeries

## Abstract

Symptomatic thoracic disc herniation (TDH) is a rare entity, accounting for less than 4% of all surgically treated disc herniations, yet it can produce devastating myelopathy because of the precarious vascular supply and limited anatomic reserve of the thoracic spinal cord. Traditional open approaches, such as transthoracic discectomy and costotransversectomy, are effective but carry substantial approach-related morbidity. Full-endoscopic Interlaminar thoracic discectomy has recently emerged as a minimally invasive alternative that allows ventral cord decompression through a small skin incision, with reduced blood loss, shorter hospital stay, and the option of awake surgery. We report the case of a 58-year-old man who presented with progressive mid-back pain, lower-extremity weakness, gait instability, and a sensory level at T10. Magnetic resonance imaging (MRI) and computed tomography (CT) demonstrated a right paracentral soft disc herniation at the T1-T2 level with significant ventral cord compression. The patient underwent a full-endoscopic interlaminar thoracic discectomy under general anesthesia. Postoperative imaging confirmed complete decompression of the spinal cord, and the patient experienced rapid neurological recovery with resolution of mid-back pain and substantial improvement in lower-extremity strength and gait at six-month follow-up. This case adds to the growing evidence supporting full-endoscopic discectomy as a safe and effective treatment option for selected patients with symptomatic TDH and highlights the technical feasibility of ventral cord decompression through an interlaminar endoscopic corridor.

## Introduction

Thoracic disc herniation (TDH) is uncommon compared with cervical and lumbar disc disease, with a reported annual incidence of approximately one per million and accounting for only 0.15-4% of all symptomatic disc herniations requiring surgical intervention [1,2]. Modern imaging has revealed asymptomatic TDH in 11-37% of the general population, but only a small subset develops symptoms severe enough to warrant surgery [1]. The condition typically affects middle-aged adults, with most lesions occurring below the T8 level, where increased segmental mobility and biomechanical inflection forces predispose the disc to degeneration and herniation [2,3].

The clinical presentation of TDH is highly variable and frequently nonspecific, often producing a delay in diagnosis [1,3]. Patients may present with axial mid-back pain, radicular thoracic pain mimicking visceral disease, sensory disturbances, and progressive myelopathic signs such as gait dysfunction, lower-extremity weakness, hyperreflexia, and bowel or bladder dysfunction [1,3,4]. The combination of a kyphotic spinal curvature, narrow thoracic canal, watershed cord vasculature, and tethering by the dentate ligaments and ribs makes the thoracic spinal cord particularly vulnerable to even minor intraoperative manipulation [4,5].

The surgical history of TDH reflects this anatomic challenge. Early laminectomy series produced unacceptably high rates of paraplegia, prompting the development of posterolateral approaches (transpedicular, transfacet, costotransversectomy, and lateral extracavitary) and ventral approaches (open transthoracic and thoracoscopic) [5-7]. Although these techniques are effective for cord decompression, they are associated with significant approach-related morbidity, including pulmonary complications, intercostal neuralgia, cerebrospinal fluid (CSF) leakage, and the frequent need for instrumented fusion [6,7]. A recent systematic review and meta-analysis of more than 3,000 patients undergoing traditional thoracic discectomy reported a pooled perioperative complication rate of 25% and a reoperation rate of 7%, with mean blood loss exceeding 500 mL and a mean hospital stay of seven days [8].

Full-endoscopic spine surgery has steadily expanded from its original lumbar applications to the cervical and thoracic regions [9-11]. Telfeian and Wagner described the modern technique of full-endoscopic transforaminal thoracic discectomy, which allows the surgeon to access ventral thoracic disc pathology through a small paraspinal incision under continuous saline irrigation and direct visualization, often with the patient awake [9,10]. Reported advantages include preservation of the posterior bony elements, minimal soft-tissue dissection, reduced blood loss, shorter hospital stay, and lower rates of approach-related complications, while still permitting ventral cord decompression [11-13]. Here we report our experience with a patient who underwent full-endoscopic transforaminal discectomy for a symptomatic (T1-T2) disc herniation with thoracic myelopathy, with a focus on the operative technique, imaging findings, and clinical outcome.

## Case presentation

A 58-year-old man presented to our outpatient spine clinic with a two-week history of progressively worsening mid-back pain associated with bilateral lower-extremity weakness, gait instability, and a band-like sensation around the lower trunk. The patient also reported intermittent urinary urgency without frank incontinence. There was no preceding history of significant trauma, malignancy, intravenous drug use, or constitutional symptoms. Conservative management with oral analgesics, physiotherapy, and activity modification over the preceding month had failed to produce sustained relief, and the patient described progressive functional decline with difficulty climbing stairs and frequent falls in the weeks before presentation.

On general examination, the patient was hemodynamically stable with no spinal deformity. Neurological examination revealed a grade 3/5 weakness in both lower extremities, predominantly affecting hip flexion and knee extension, with preserved upper-extremity strength. There was a reproducible sensory level to pinprick and light touch at approximately T1\T2 bilaterally. Deep tendon reflexes were brisk in the lower extremities (3+) with bilateral sustained ankle clonus and an upgoing plantar response (positive Babinski sign), consistent with thoracic myelopathy. Tandem gait was unsteady, and the modified Japanese Orthopedic Association (mJOA) score for the thoracic spine was 8/11 preoperatively. Visual analog scale (VAS) scores were 7/10 for axial back pain and 4/10 for radicular thoracic pain.

An MRI of the thoracic spine demonstrated a left paracentral soft disc herniation at the T1-T2 level producing significant ventral compression of the spinal cord, with effacement of the anterior CSF space and focal cord signal change on T2-weighted sequences (Figures 1, 2).

**Figure 1:**
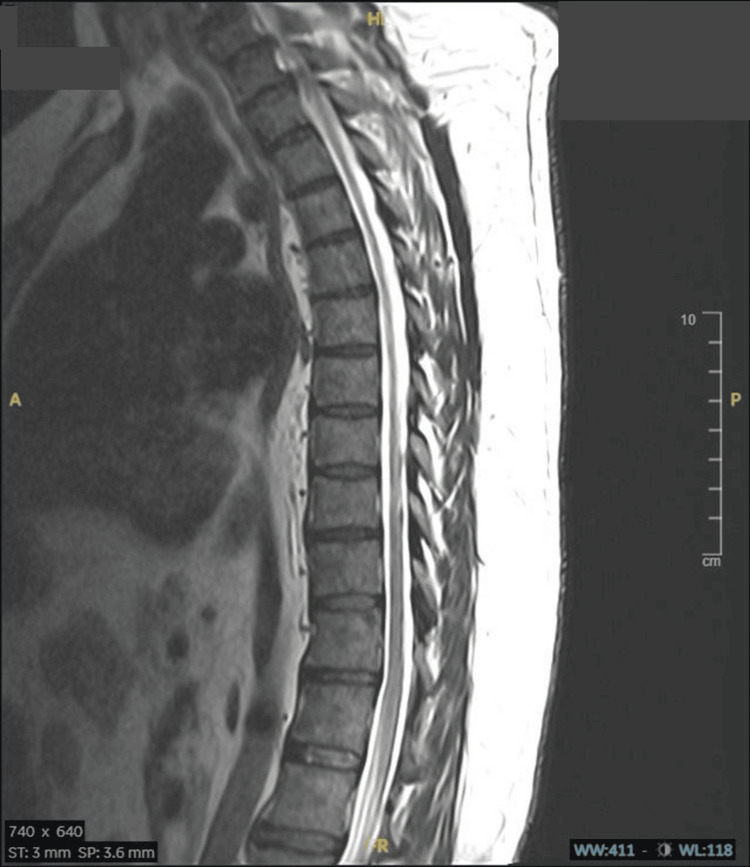
Sagittal T2-weighted MRI showed a central-to-left paramedian disc herniation at T1–T2, causing ventral spinal cord compression and effacement of the anterior subarachnoid space. MRI: Magnetic resonance imaging

**Figure 2:**
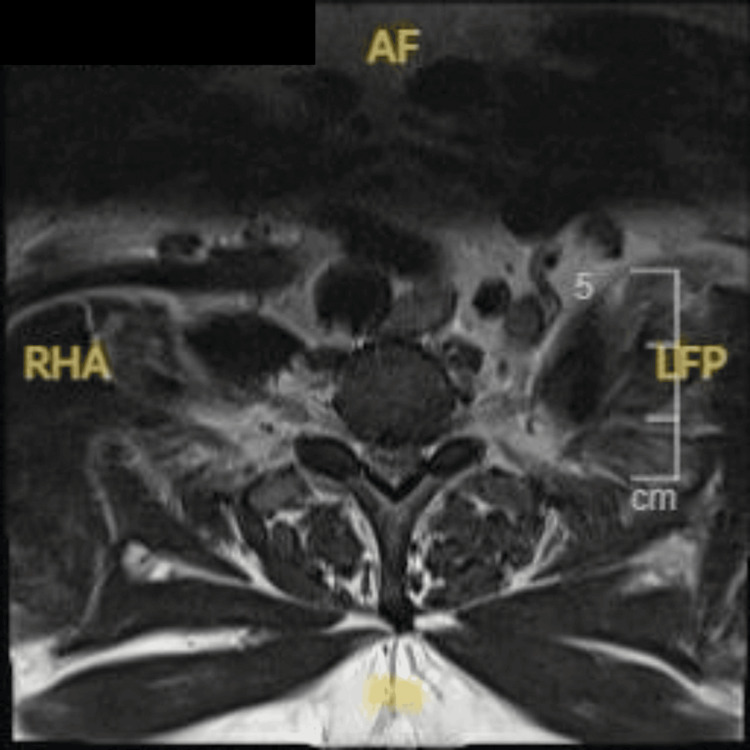
Axial T2-weighted MRI confirmed left-dominant disc protrusion. MRI: Magnetic resonance imaging

After multidisciplinary discussion and detailed informed consent, including a comprehensive review of open and minimally invasive surgical alternatives, the patient elected to proceed with full-endoscopic interlaminar thoracic discectomy. Surgery was performed in the prone position under general anesthesia. The skin entry point was planned approximately 6-8 cm lateral to the midline on the symptomatic side using anteroposterior and lateral fluoroscopy, with the trajectory aimed at the safe triangular working zone of the T1-T2 foramen, just superior to the cranial border of the lower pedicle and ventral to the rib head.

Following infiltration with local anesthetic, an 18-gauge spinal needle was advanced under biplanar fluoroscopic guidance to the lateral border of the foramen (Figure 3). A guidewire was then inserted, followed by sequential dilators and the working cannula. Foraminoplasty was performed using endoscopic burrs and Kerrison rongeurs to undercut the superior articular process and the cranial portion of the rib head, thereby enlarging the foramen and creating a safe ventral working corridor without entering the pleural cavity. Continuous saline irrigation provided clear visualization throughout the procedure. The herniated disc fragment was identified ventral to the dura, mobilized away from the cord with a blunt probe, and removed piecemeal using endoscopic graspers (Figure 4). Bipolar radiofrequency was used judiciously for hemostasis. After complete removal, the dura was inspected and observed to pulsate freely, confirming adequate decompression. The working cannula was then withdrawn, and the single skin incision was closed with a subcuticular suture. Total operative time was approximately 90 minutes, and estimated blood loss was minimal (less than 30 mL).

**Figure 3:**
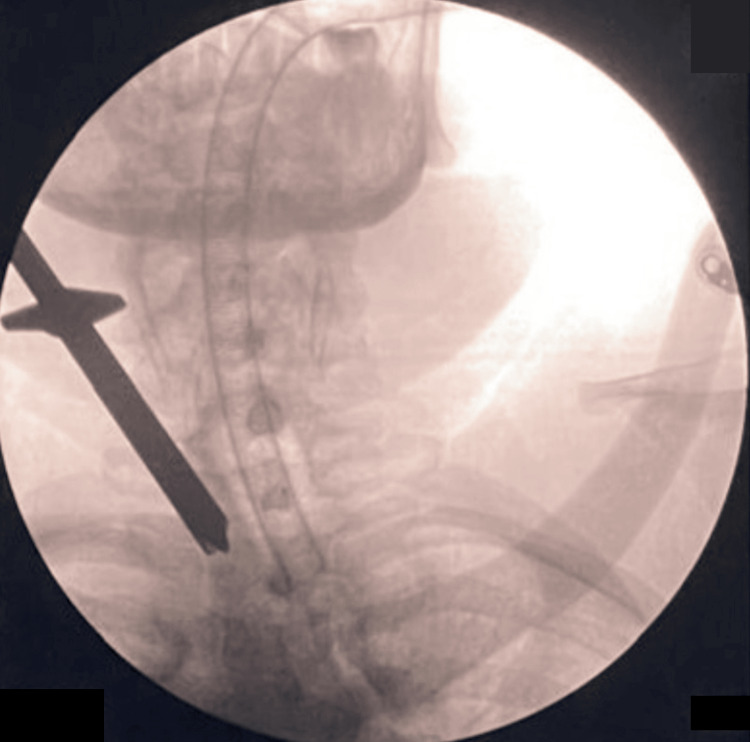
Intraoperative fluoroscopy showing endoscopic working cannula trajectory targeting the T1–T2 disc space via left intralaminar approach.

**Figure 4:**
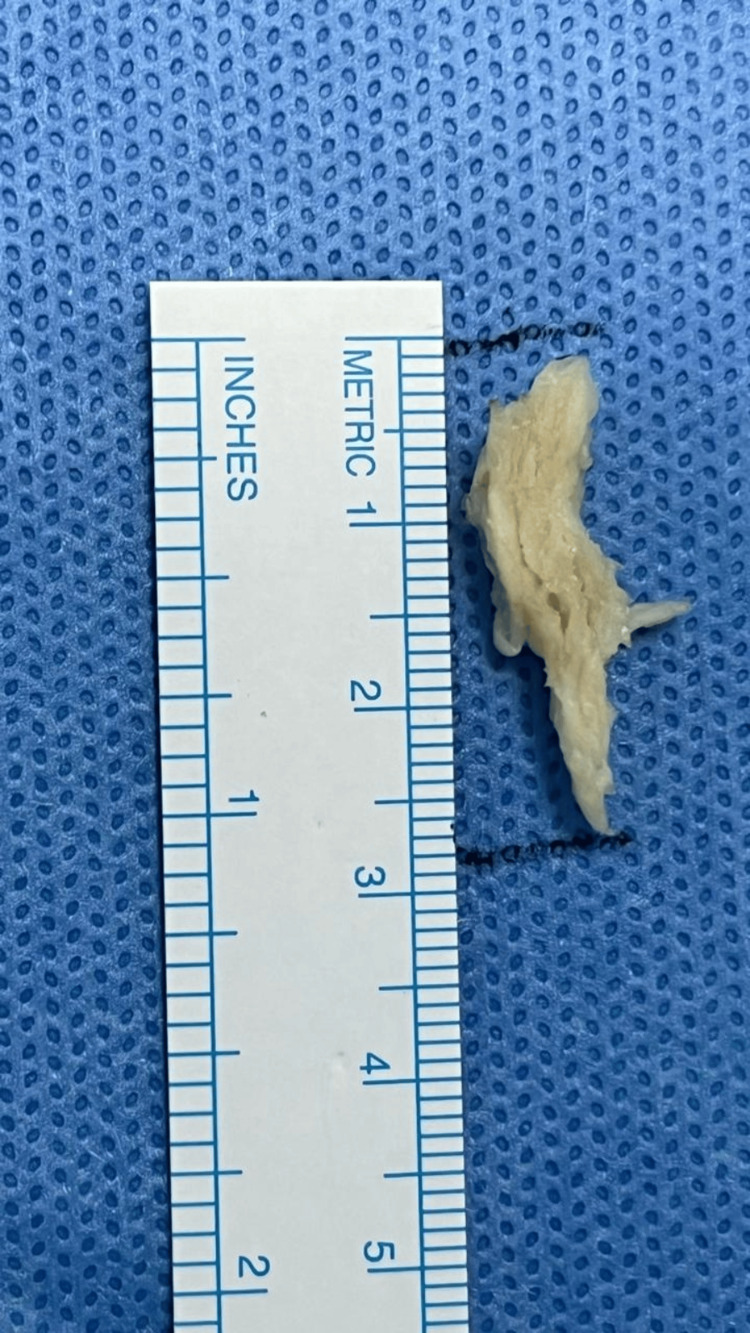
Extracted herniated disc fragment measuring approximately 2.5 cm.

The postoperative course was uneventful. The patient was mobilized within hours of surgery and reported immediate resolution of the band-like thoracic radicular pain. There were no perioperative complications, including no dural tear, no pneumothorax, and no new neurological deficit. The patient was discharged on the third postoperative day. At the three-month and six-month follow-up visits, the patient demonstrated marked improvement, with the VAS for axial back pain decreasing from 7/10 to 1/10 and the mJOA score improving from 8/11 to 11/11. Lower-extremity strength returned to grade 5/5, gait was normal, and urinary urgency had resolved. Postoperative MRI obtained confirmed complete removal of the herniated fragment with restoration of the ventral CSF space and resolution of the cord compression (Figures 5, 6). Postoperative CT demonstrated the limited bony resection of the foraminoplasty, with preservation of the pedicle, lamina, and facet joint integrity, and no evidence of segmental instability (Figure 7).

**Figure 5:**
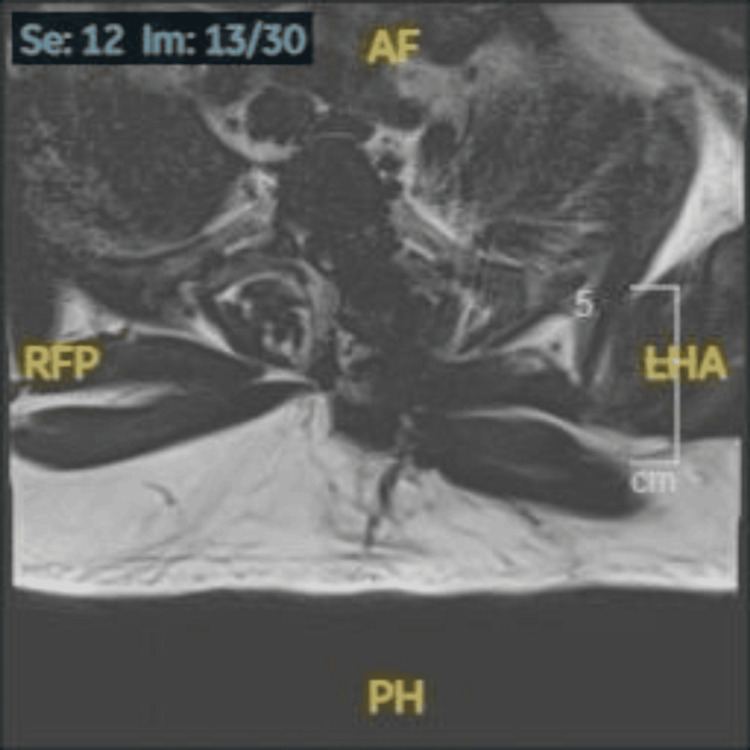
Postoperative sagittal T2-weighted MRI confirming resolution of ventral cord compression and normal spinal alignment. MRI: Magnetic resonance imaging

**Figure 6:**
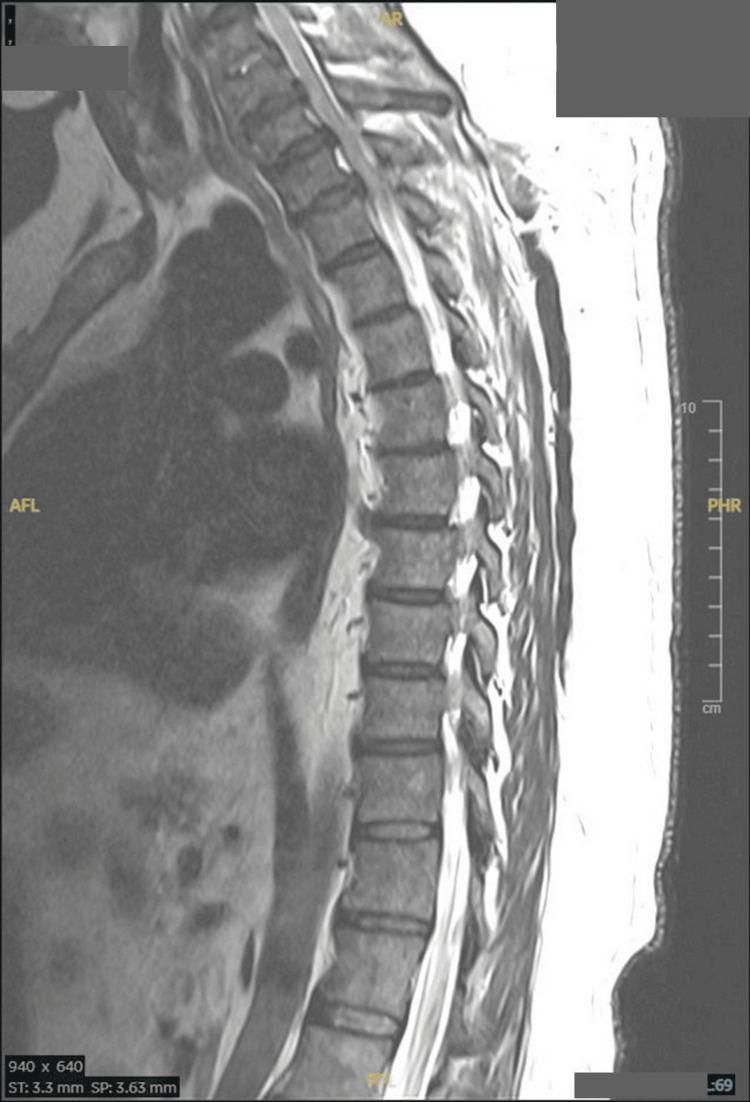
Postoperative axial T2-weighted MRI at T1–T2 level showing restoration of anterior CSF space and decompressed spinal cord. MRI: Magnetic resonance imaging, CSF: Cerebrospinal fluid

**Figure 7:**
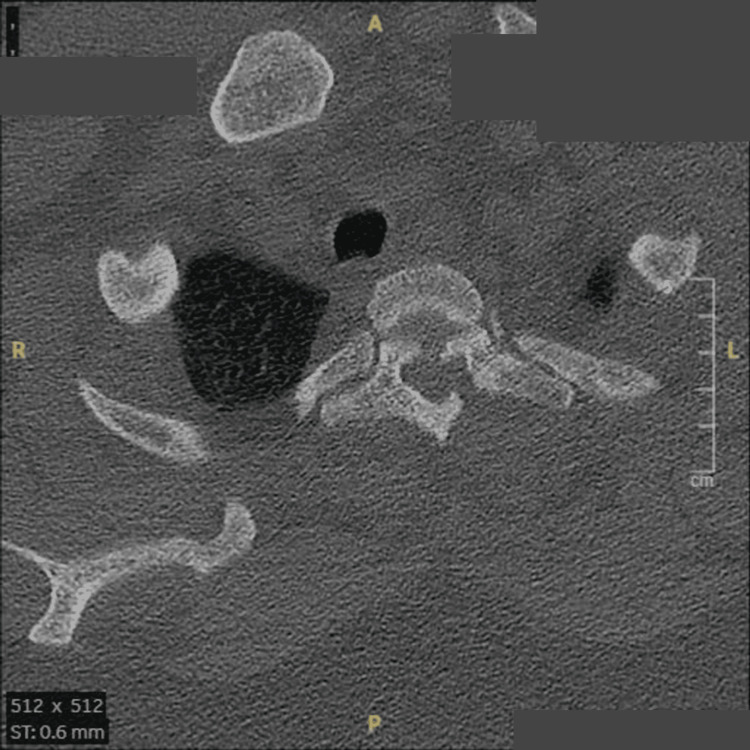
Postoperative axial CT showing left T2 laminotomy.

## Discussion

Symptomatic TDH remains one of the most challenging conditions in spine surgery because of the unique anatomic and biomechanical constraints of the thoracic spinal cord. The combination of a narrow canal, kyphotic alignment, watershed vascular supply, and limited tolerance to retraction means that even small adjustments in surgical strategy can have major neurological consequences [4,5,7]. Decompressive laminectomy alone has long been abandoned for ventrally placed thoracic discs because of its unacceptably high rate of neurological deterioration, and the modern algorithm relies on either ventrolateral access (open transthoracic or thoracoscopic) or posterolateral corridors (transpedicular, transfacet, costotransversectomy, or lateral extracavitary), depending on the location and consistency of the herniation [5-7,14].

Although these traditional approaches achieve adequate decompression in most patients, they carry a non-trivial morbidity burden. Sofoluke and colleagues, in a multicenter analysis of more than 3,000 patients undergoing traditional thoracic discectomy, reported a pooled perioperative complication rate of 25%, a reoperation rate of 7%, and frequent need for instrumented fusion (37%), with mean blood loss exceeding 500 mL and a hospital stay averaging seven days [8]. Pulmonary complications, intercostal neuralgia, CSF leak, and approach-related pain are well-documented sequelae of transthoracic surgery, while extensive bony removal in posterolateral approaches may necessitate fusion to prevent iatrogenic instability [6,7,14]. These considerations have driven the steady migration toward minimally invasive options.

Full-endoscopic transforaminal thoracic discectomy was first described in detail by Wagner and Telfeian and has subsequently been refined into a reproducible technique [9,10,15]. The transforaminal corridor exploits the natural opening between the rib head, pedicle, and superior articular process, allowing the surgeon to reach the ventral epidural space without requiring extensive bony resection or entry into the thoracic cavity [10,11]. The angled endoscopic camera provides an unobstructed view of the ventral dural surface, which is particularly advantageous for centrally located herniations that would otherwise demand a transthoracic approach [9-11]. Continuous saline irrigation maintains a clear operative field, and the use of a single small skin incision dramatically reduces soft-tissue trauma. In selected cooperative patients, the procedure can even be performed under local anesthesia with conscious sedation, offering real-time neurological feedback as a form of intraoperative monitoring [9,16].

A growing body of evidence supports the safety and efficacy of full-endoscopic thoracic discectomy. Silva et al. performed a single-arm meta-analysis of 13 studies including 285 patients and reported low pooled rates of dural tear, recurrent herniation, and persistent myelopathy, with significant improvement in neurological function across the cohort [11]. Komp and Ruetten described the use of a full-endoscopic anterior approach for giant and calcified TDHs, achieving favorable outcomes even in technically demanding cases [12]. Gao and colleagues reported that the modified transforaminal technique with foraminoplasty resulted in a mean improvement in the mJOA score from 7.4 to 10.2, with no perioperative complications in their series [17]. The patient described in the present report demonstrated a clinical course consistent with these published outcomes, including rapid resolution of axial and radicular pain, marked improvement in mJOA score, and discharge on the first/second postoperative day.

Despite these advantages, the technique is not without limitations. The transforaminal corridor is technically demanding and is associated with a steep learning curve, and complications such as dural tear, dysesthesia, hematoma, and incomplete decompression have been reported, particularly during a surgeon’s early experience [13,18]. Heavily calcified or transdural herniations remain a relative contraindication for many surgeons, and conversion to an open approach must always be feasible [12,13]. Strict patient selection, meticulous preoperative planning with both MRI and CT, and proficiency in fluoroscopic and endoscopic anatomy of the thoracic foramen are essential to minimize complications [10,13,18]. Adequate training in lumbar endoscopic surgery is generally regarded as a prerequisite before progressing to thoracic indications [19,20].

## Conclusions

Full-endoscopic Interlaminar thoracic discectomy is a safe and effective minimally invasive option for the treatment of selected patients with symptomatic TDH. In the case presented, the technique enabled complete ventral cord decompression through a single small skin incision, with negligible blood loss, an uneventful postoperative course, and rapid neurological recovery. As experience with full-endoscopic spine surgery continues to grow, this approach is likely to assume an increasing role in the surgical armamentarium for thoracic disc disease, although careful patient selection, structured training, and thoughtful comparison with traditional techniques remain essential. Larger comparative studies and longer-term follow-up are needed to define the precise indications, learning curve, and durability of clinical outcomes.
